# Quantitative biology of single neurons

**DOI:** 10.1098/rsif.2012.0417

**Published:** 2012-08-22

**Authors:** James Eberwine, Ditte Lovatt, Peter Buckley, Hannah Dueck, Chantal Francis, Tae Kyung Kim, Jaehee Lee, Miler Lee, Kevin Miyashiro, Jacqueline Morris, Tiina Peritz, Terri Schochet, Jennifer Spaethling, Jai-Yoon Sul, Junhyong Kim

**Affiliations:** 1 Department of Pharmacology, Perelman School of Medicine, University of Pennsylvania, 36th and Hamilton Walk, Philadelphia, PA 19104, USA; 2 Department of Biology, School of Arts and Sciences, University of Pennsylvania, 301 Lynch Laboratories/6018, 433 S. University Avenue, Philadelphia, PA 19104, USA; 3 PENN Genome Frontiers Institute, University of Pennsylvania, 301 Lynch Laboratories/6018, 433 S. University Avenue, Philadelphia, PA 19104, USA

**Keywords:** quantitative biology, single neurons, single cells, transcriptomics, proteomics, splicing

## Abstract

The building blocks of complex biological systems are single cells. Fundamental insights gained from single-cell analysis promise to provide the framework for understanding normal biological systems development as well as the limits on systems/cellular ability to respond to disease. The interplay of cells to create functional systems is not well understood. Until recently, the study of single cells has concentrated primarily on morphological and physiological characterization. With the application of new highly sensitive molecular and genomic technologies, the quantitative biochemistry of single cells is now accessible.

## 1. Introduction

In a time when the study of science was the imprimatur of educated individuals, people turned their attention to the sky above through the study of astronomy and to our natural environment through the study of biology. Direct observational biology was the foremost method for scientific discovery. Biological classification relied heavily upon direct observation of minute but macroscopic features. Advances in astronomy lent biologists improved optics which allowed finer, more detailed magnified observations of objects in their everyday world.

In 1665, the Royal Society published Robert Hooke's treatise ‘Micrographia’. Its first run of 2800 copies sold out within 2 years of its initial publication, making it the first scientific bestseller. In a series of chapters entitled ‘Observations’, this text presented strikingly detailed drawings and descriptions of Hooke's observations of shells and the such as made using his version of the newly developed compound microscope. Notably, in Observation 18, Hooke provided the first detailed description of the structure of plants through his observations of cork, wherein he described the observed small boxes as cells.

‘I took a good clear piece of Cork, and … cut off … an exceeding thin piece of it, and placing it on a black object Plate, because it was it self a white body, … I could exceeding plainly perceive it to be all perforated and porous … these pores, or cells, … were indeed the first microscopical pores I ever saw, and perhaps, that were ever seen, for I had not met with any Writer or Person, that had made any mention of them before this.’

This was the first use of the word ‘cell’ to describe a biological entity. Anton Leeuwenhoek, a Dutch biologist, described the first living cell. In a series of letters first published in 1676 and spanning a decade of work, Leeuwenhoek's observations were presented to the Royal Society [1], wherein he called the single-celled organisms ‘animalcules’. The initial description of a single-celled organism was met with scepticism, but was eventually accepted, resulting in his appointment as a Fellow of the Royal Society in 1680.

These initial observations were expanded over the next 150 years, resulting in the elaboration of the cell theory by Theodor Schleiden and Jakob Schwann, with the addition of the important contribution of Rudolf Virchow producing what is known as the Modern Cell Theory [2]. The basic tenets of the theory state that the cell is the fundamental unit of structure and function for living organisms, all living things are made up of one or more cells and that all cells arise from pre-existing cells. The codification of the Modern Cell Theory dramatically reduced the reliance of science upon the idea of vitalism heralding the dawn of modern biology and paving the way for acceptance of Darwin's theories of evolution.

Biological investigation at the single-cell level has advanced over time, resulting in recognition of the existence of millions of different cell types ranging from bacteria through plants to eukaryotic cells. Identification of cell type has traditionally occurred through morphological and functional phenotyping. The advent of modern genomics and imaging technologies, such as the introduction of microscopy during the time of Hooke and Leeuwenhoek, has heralded a revolution in our understanding of cells and their functional identity. In this review, we will present some of the modern work on single cells, discuss insights provided by this work and suggest future directions for development of single-cell analysis.

## 2. Rationale for the study of single cells

One of the hallmarks of multicellularity is the specialization of cells for the performance of different tasks, with these cells organized into a hierarchy of functionally and morphologically distinct, but genetically identical, organ systems and tissues [3]. Inspection of a tissue cross section with the aid of a strong microscope and contrast staining would reveal cells with distinct characteristics, specific to the tissue type from which they derive. Neural tissue would reveal a network of glial cells and neurons, with long projections that interconnect to facilitate the propagation of signals. Heart tissue would reveal stacks of filamentous cardiomyocyte cells, while blood would be composed of smooth, concave erythroid cells intermixed with irregularly shaped leucocytes and platelet cells. These differences are the phenotypic consequences of a divergent set of biochemical reactions occurring within each cell, mediated by a specific recipe of biomolecules—proteins and RNA—produced from differential expression of the genes in the organism's genome.

**Figure 1.**
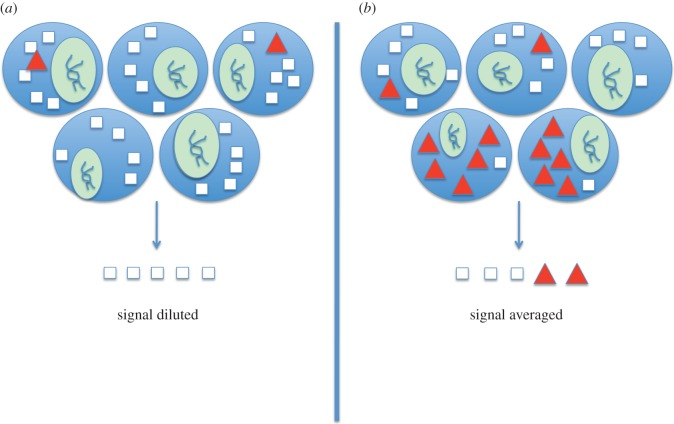
Loss of single-cell resolution is endemic to tissue-level analyses. Loss of resolution can be due to: (*a*) signal dilution, in which a lowly expressed biomolecule (red triangles) fails to be detected owing to the predomination of other species (white squares) in the aggregate sample; or (*b*) signal averaging, in which the biologically relevant ratios of biomolecular species are inaccurately represented in the aggregate sample (total number of a particular mRNA divided by the number of cells). The nucleus of the cell is depicted in the green oval situated in each cell.

To effectively study and understand biological phenomena at the molecular level, cellular variability must be accounted for. However, this can be difficult to achieve using standard biochemical techniques, which vary in their ability to detect and quantify single-cell quantities of biomolecules. Typical tissue-level or cell-population-level analyses often result in the loss of cellular resolution and context (figure 1). On the one hand, the source material may be dominated by a large number of cells that do not express a biomolecule of interest, resulting in a dilution of the signal below the lower detection limit of a technique. On the other hand, cell populations can average out individual cellular co-expression patterns, making it difficult to determine, for example, whether two biomolecules always occur within the same cell or occur with mutual exclusion. Thus, targeted single-cell analyses are needed to understand cell-to-cell variability, and ultimately, how subtle differences in cellular phenotype induce biological phenomena such as learning and memory, and how cell-specific changes lead to dysfunction, as in cancer.

## 3. Variability and stochastic events in gene expression

Although cells of the same type share many distinguishing characteristics, they are generally not identical. Living cells are not static—they consume metabolites, receive external stimuli, adapt to changing microenvironments, grow and divide. All of these processes are mechanistically grounded in transient or even permanent gene expression changes that are generally not uniform across all cells. Sensory neurons, for example, can occur in complex distributions to allow an organism to respond to stimuli with spatial specificity [4,5]. A discrete stimulus will involve the cascading activation of many, but not all neurons, thus inducing cell-variable physiological effects and gene expression changes [6]. Evidence suggests that gene expression changes underlie the generation of long-term memory [7], which is thought to involve the enhancement of specific synaptic connections between individual neurons [8] resulting in experience-dependent neuronal heterogeneity.

Even in the absence of explicit environmental differences, cell-to-cell variation would still exist. This is due to the inherent stochasticity of gene expression, which requires physical interaction between a relatively small number of molecular players. When few molecules of reactants exist in a closed volume, as in a cell, time to interaction is random, resulting in variation between cells in terms of when, and if, a given gene is expressed [9–12].

Stochastic gene expression is often subdivided into two categories, by whether the noise is due to random interaction at the promoter or transcript of a given gene (intrinsic variation), or whether the source of noise is variation in regulatory molecules (extrinsic variation) [9]. These two classes produce different stereotypic behaviours. Elowitz *et al*. designed a reporter system to monitor these different types of noise, inserting cyan and yellow fluorescent genes into *Escherichia coli,* each attached to identical promoters. Intrinsic noise, or variations due to random binding of a promoter or transcript, is observed as differences in the expression of the two fluorescent signals within a given cell. Variation in regulatory molecules or extrinsic noise, such as transcription factors, polymerases and ribosomes, results in consistent expression of these two copies within one cell, but observed variation between different cells [9]. These two types of variability occur on different timescales with intrinsic noise fluctuating at the rate of transcription, while extrinsic noise persists for the life of the regulatory machinery. Both the timescale of noise and a cell's environment (e.g. in a single-cell or multicellular organism) interact to produce phenotypic and fitness consequences.

Because gene expression underlies the physical characteristics of a cell or organism, random variability in transcription may result in molecular misregulation or in variable phenotype among genetically and developmentally identical cells. This generation of difference may either be harmful or beneficial to an organism, and regulatory circuits may have evolved to reduce or amplify noise respectively [13,14]. Random over-expression of a gene can result in wasted molecular resources, while random under-expression may reduce the efficiency of the cell for the activity of a given gene product [14]. All of these cases may incur a fitness cost. The extent of intrinsic noise in gene expression is a function of relative rates of transcription, translation, as well as mRNA and protein degradation. Ozbudak *et al*. [15] demonstrate that efficient transcription and less-frequent translation result in minimal variability in protein abundance in prokaryotes. In eukaryotes, the rates of transition between active and inactive DNA significantly affects noise, with rapid transitions followed by slow transcription presumptively producing less variability [16]. Higher gene copy number decreases noise, as do negative autoregulatory feedback circuits [16]. These observations suggest genetic or evolvable control of variability in gene products.

Phenotypic differences between genetically and developmentally identical cells can also be useful to an organism, as they facilitate phenotypic plasticity. Two prime strategies related to beneficial aspects of this phenomenon are bacterial ‘bet-hedging’ and multicellular development [17]. ‘Bet-hedging’ allows an isogenic bacterial population to survive rapid environmental changes, with some proportion of the population randomly exhibiting an alternate phenotype. As reviewed by Eldar and Elowitz, even in good growth conditions, a sub-population of a larger *Bacillus subtilis* population remains in a ‘competent state’, without DNA replication or cell division, at any given time. If the food supply suddenly becomes constricted, these non-dividing cells are better suited to survive. When food again becomes abundant, this population regenerates its initial diversity, with the majority of cells metabolizing food and dividing. This binary state results randomly from noise in the expression of a key regulator, variation that is amplified and stabilized by the structure of the gene regulatory circuit. Positive autoregulation of ComK, a gene responsible for competence, amplifies random instances where its own expression exceeds a threshold value, creating a switch that results in biphasic gene expression and the observed binary phenotype.

Multicellular organisms may directly use stochastic mechanisms to generate cell-to-cell variability when deterministic regulation required to produce the desired spectrum of phenotypes would be exceedingly complex [18]. Leveraging stochastic mechanisms allows for the generation of significant diversity without hard-wiring regulatory circuits to produce all possible outcomes. Such a stochastic mechanism has been hypothesized to underlie certain kinds of neuronal diversity. In the olfactory system, sensory neurons contain over 1000 unique odour receptors, each expressed with only one specific receptor expressed per cell [19]. Some authors have suggested that these neurons may be generated through a positive feedback mechanism that amplifies gene expression noise to produce on–off expression of an odorant receptor gene, coupled with a negative feedback mechanism that represses all other receptors [20]. With a probability distribution for activation across odour receptor genes, this system can generate a population containing the diverse spectrum of receptors observed.

Although gene expression variation is perhaps the easiest to imagine in the process of transcription and translation, random variation also arises in other regulatory processes, including isoform generation, allele-specific expression, chromatin states, molecular partitioning at cell division and signalling cascades [13,20–23]. As in the above cases, the structure of regulatory circuits can suppress or amplify this noise, depending on whether the resulting variability has been beneficial over evolutionary time.

Most single-cell studies to date that have characterized and quantified variability have been performed in prokaryotic systems, which, from a technical perspective, are easily accessible single-cell systems. Single-cell studies in multicellular organisms and more complex heterogeneous tissues, such as brain tissue, present various technical obstacles. While a number of techniques, such as imaging and electrophysiology, can readily assess single cells, other techniques, such as transcriptome and proteome analysis, are limited by the amounts of input material from a single cell. In the following text, we review various attempts and technical improvements that have developed over the past decade to facilitate the study of single cells.

## 4. Microscopy techniques and visualization of single cells

Since the time of Hooke and Leeuwenhoek, microscopy has come a long way; cells can now be visualized in intact tissue with nanometre and three-dimensional resolution, making it possible to investigate single-cellular compartments and single molecular interactions in great detail. The fundamental structure and principles of compound microscopy are the same as from Hook's era, although progress in electronics and computer science have greatly enhanced the sensitivity of microscopy for signal-to-noise detection and image resolution [24]. For example, bright sunlight was first replaced with a white light source, and has now been supplanted by lasers. Detection of magnified objects with the human eye has been replaced with cameras and specialized photon-detecting apparatus. In the past decade, progress in microscopy has focused on generating higher resolution images of anatomical structures, improving spectral separation of emitted light for simultaneous detection of multiple dyes and higher temporal speed for faster image acquisition. The availability of multiple light sources such as laser lines for scanning microscopes and light emitting diodes for epifluorescence microscopes have expanded the choice of available fluorochromes and opened up the possibility of synthesis of wavelength-specific fluorochromes in the future [25].

The conventional method of using spectral filters has limitations in separating multiple, closely located emission spectra and, in the case of low-level fluorescence detection, is especially sensitive to instrumental artefacts such as dark-current (electronic noise) produced by the detector [26]. Currently, microscopy using advanced spectral detection methods are based on scanning through the emission spectra or static capture of the entire emission range with multiple detector arrays for each wavelength [27]. Both methods are capable of distinguishing the difference from FITC and EGFP, which have very similar emission spectra, separated by only about 20 nm. As the emissions are dispersed en route to the detector, the number of detectable photons from each pixel for any specific wavelength is also reduced. However, this limitation can be overcome by choosing efficient fluorochromes with better quantum efficiency and extinction coefficients, by using dyes that produce bright emissions, and by using more efficient detectors such as a GaAsP photomultiplier tube or electron multiplying charge-coupled device. The optimal combination of fluorochrome and detector with spectral deconvolution allows the use of multiple fluorescent reporter proteins with multiple physiological indicators at the same time. Currently, the typical limits of conventional microscopy allow three channels of detection, whereas these technologies should extend the number of possible channels to at least six.

Using different lenses or small scanning area (zoom), microscopy can be used as a non-destructive investigative tool for studies of samples ranging from a single cell in tissue through the resolution of subcellular compartments in live samples. The recent development of a miniaturized microscope that can be mounted on a mouse head allows monitoring of brain activity with single-cell resolution while the animal is freely moving [28,29]. These advances allow us to move forward to a more comprehensive understanding of the biology of living organisms in action.

In order to achieve the highest resolution for the study of cellular anatomy, electron microscopy is the best approach. Recent serial section scanning electron microscopy and digital reconstruction have been used to demonstrate three-dimensional ultrastructure of the cell [30]. Although EM is costly in terms of both time and labour, the combination of EM and fluorescence microscopy also demonstrates the potential future application of correlating single-cell proteomics to determine protein function with highly sensitive imaging to determine localization [31]. Using various fluorescence indicators and proteins, the identification of single cells in tissue and monitoring of physiological activity have become part of the daily routine for many scientists. Since Abbe's definition of the physical limitation of resolution in far-field optical systems roughly 130 years ago, many scientists have devoted their efforts to overcoming the ‘Abbe's diffraction limit’ [32–34]. Recent advances in microscopy achieve nanometre-level resolution using engineered proteins or physical properties of fluorescence molecules to successfully visualize subcellular structures beyond the limit of diffraction of light [35,36]. Depending on the usage of fluorescence proteins or illumination methods, the microscopy technology can be divided as photoactivated localization microscopy [36], fluorescence photoactivation localization microscopy [37], stochastic optical reconstruction microscopy [38], which work by switching the fluorescence proteins on and off, or by methods using manipulation of illumination methods such as stimulated emission depletion [35], ground state depletion [39] and structured illumination microscopy [40,41]. All of these available super-resolution microscopy techniques can be applied to live single-cell studies and bring the promise of high resolution to investigate further details of the biology of single cells that have yet to be determined by conventional techniques. Another single-cell-level, single-molecule imaging technology is fluorescence resonance energy transfer (FRET) [42]. Strictly speaking, this is not a microscopy technology but instead, usage of the microscope while taking advantage of the principles of fluorescence chemistry. Unlike conventional fluorescence microscopy using excitation and subsequent emission detection where flurochromes are chosen with separated emission spectra, FRET uses pairs of fluorochromes in which the emission of one flurochrome overlaps with the other's excitation. Detecting electron transfer between those dyes allows one to measure the conformational changes of proteins during biological activity beyond the resolution of any microscope currently available, as the signal is dependent on distances measured in angstroms. For high-resolution imaging in a single cell, using near-field illumination as excitation, total internal reflection fluorescence (TIRF) microscopy can also be used to image single cells [43]. This technology takes advantage of the property of light reflection to generate high axial resolution images of samples.

Owing to the physical properties of illumination, light takes on an hourglass shape with the smallest point centred on the object of study. This means blurring of the image is always unavoidable. Mathematical deconvolution based on the point spread function of optics has been used to address this issue and can be used to successfully reconstruct crisp images [44]. Using a pinhole in confocal microscopy, without relying on mathematical data processes, allows one to easily acquire the desired plane of focus in samples. However, in order to acquire high temporal resolution line scan in conventional confocal microscope can serve this purpose, although it produces limits on a real resolution, as only a single line can be acquired at a time. For imaging an entire cellular region, a spinning disk confocal with multiple arrays of pinholes in the optical path can acquire whole-cell-wide biological activities with high speed [45]. In addition to using physical methods of manipulating the function of light by limiting its path with pinholes or by using mathematical deconvolution, the nonlinear nature of multiphoton excitation of fluorochromes is also used to decrease blur. This method, frequently referred to as two-photon microscopy, eliminates non-specific excitation of surrounding imaging planes to generate a confocal effect. This method has been extended to *in vivo* animal imaging by using a deep penetrating long wavelength multiphoton laser, and is also commonly applied in prepared cells and tissue specimens [46]. Another advantage of the multiphoton light source is that it is relatively less phototoxic than single-photon microscopy and therefore more suitable for long-term imaging on targeted cells. The illumination of multiphoton microscopes is still bound by the diffraction limit, but owing to its defined volume and a non-descanning method for emission collection, future developments in detector design will continue to increase the precision of data that can be obtained far beyond that which can be acquired from the single-photon methods.

The use of photo-labile structures permits lasers to manipulate the cellular physiology and allow the capture of substances at the single-cell level [47,48]. Chemicals that are designed for use in either single-photon or two-photon applications are incorporated into caged compounds widely used in single-cell and subcellular functional studies. Photolysis can be produced as long as imaging is possible, and thus it can be performed at a high spatio-temporal resolution as long as the target can be simultaneously visualized. Altogether, modern microscopy allows us to identify the target single cell, monitor physiology, manipulate physiology and capture the substance responsible for, or that results from physiological activity. In the future, the automation of microscopy and higher spectral separation will allow us to image massive numbers of single cells with a variety of different biological tags. Imaging techniques provide a method whereby single cells can be visualized in real time and some cell biological parameters quantified in the context of that cell's specific natural environment.

## 5. Electrophysiology techniques and the study of ion channels in single neurons

The advent of single-cell electrophysiology was brought on by a series of elegant experiments using the squid giant axon as a model system as well as the development of the patch-clamp to gain insight into ion channel populations and signalling capabilities of single neurons [49,50]. Using a glass electrode attached to equipment capable of measuring millivolt-level changes in membrane potential or picoamp changes in ion channel currents, researchers can measure neuronal electrical responses. Electrophysiology provides a valuable tool to gain insight into how neurons and other cells respond to various electrical or pharmacological stimuli, and can be used to examine cells in culture, brain slice or *in vivo*. As electrophysiology assesses ionic flow across the plasma membrane, it can provide insight into the responsible mechanism of such currents. Although low-throughput, the ion channels contributing to signalling can be determined by understanding the ionic properties, the unique characteristics of each ion channel, and by the use of pharmacological manipulation of the cell to either inhibit, block or activate the ion channel of interest. It is now routine to test a drug's effects using single-cell recording. Variability in ion conductances can be quantified among single neurons [51–53] and when combined with other single-cell techniques, such as imaging, can generate a more complete picture of the phenotype of the investigated cell. For example, Browne *et al*. [52] performed single-cell analysis of single thalamic neurons using a combination of electrophysiology, pharmacology and single-cell PCR analysis. Differences in GABA subunit expression and pharmacological responsiveness to clorazepam, an anti-epilepsy therapeutic, were demonstrated among single cells. One of the most promising findings was that single-cell differences in subunit expression were correlated with differential pharmacological responsiveness suggesting subunit-defined specificity to clorazepam responsiveness. Combining electrophysiological studies of function with single-cell RNA profiling techniques and imaging provides an integrative analysis of live functioning cells. Such integrative approaches promise to highlight the molecular underpinnings of cellular physiology and functioning.

## 6. *In situ* hybridization techniques and RNA detection in single cells

Similar to electrophysiology, *in situ* hybridization (ISH) is a low-throughput method that allows the study of single cells. ISH is an imaging technique used to investigate the number and identity of RNA species in single cells, and it can be performed on cells or tissue sections without isolation or manipulation of endogenous RNA. This first method of visualizing nucleic acids was established in the early 1970s on the basis of the use of radioactive material to label oligonucleotide probes [54]. Ten years later, fluorescence *in situ* hybridization (FISH) advanced the technique of ISH as it presented many advantages in resolution, speed and safety [55]. FISH is performed by hybridizing a labelled nucleic acid probe (either DNA, RNA or modified nucleic acids) on a complementary target in fixed and permeabilized tissue or culture samples. Despite having a lower sensitivity than other methods, ISH is one of the few methods that can provide subcellular localization information and can simultaneously show variability between cells and within subcellular regions. Although the basic principles of FISH have remained unchanged, high-sensitivity detection, assaying multiple mRNA species simultaneously, and automated data collection and quantitative analysis have advanced the field significantly. One caveat to the use of ISH or FISH is that a limited number of targets can be investigated simultaneously and that the use of labelled probes allows targeting only the specific region of the transcript selected *a priori* [56]. In general, successful FISH results are achieved when the concentrations of the target sequence provide enough contrast above the background fluorescence.

Many advances in this procedure have been introduced to avoid the limitation of low-throughput and to allow a more refined target detection, sensitivity and resolution. For instance, the use of oligoprobes labelled with five fluorochromes allowed the detection of single molecules present at low concentration within a cell with binding of only one oligonucleotide probe [57]. This technique provided an advancement in sensitivity of up to two orders of magnitude over what was previously possible [58]. Another improvement in FISH is multiplex labelling of different RNA species within a single cell. In this method, several dyes are conjugated to oligonucleotide probes directed to different RNAs and allowed to simultaneously visualize several transcription sites active at the time of tissue harvesting [59]. This modification of FISH has highlighted the presence of extensive variability in single-cell gene expression. Variations in *in situ* labelling techniques are continuously introduced and improved on providing a different level of detection from which to choose. The specifications, sensitivity and resolution of these techniques are thoroughly reviewed elsewhere [60–62].

Significant progress in computer-automated data processing has enabled researchers to analyse FISH imaging data at a much larger scale. For instance, a recent FISH study in *Drosophila* embryos showed that the majority of mRNAs (71% of 3370 genes investigated) were asymmetrically localized, highlighting the fact that targeted mRNA localization is a widespread phenomenon occurring at multiple developmental stages and across phyla [63,64]. FISH has now been developed and used for a variety of organisms and cell types. For single-celled organisms such as bacteria and yeast, detailed analyses of gene expression patterns with FISH have revealed the bursting nature of gene transcription [65–67] in these organisms.

## 7. Subcellular mRNA localization patterns can be visualized using fish

Subcellular RNA localization has been conserved throughout evolution and has been described in a large variety of species ranging from yeast to mammals. Variation in subcellular distribution occurs in dividing cells such as fibroblasts as well as in post-mitotic cells such as neurons, and is used by the cell to target classes of RNAs to subcellular sites where they function, e.g. mRNA to direct protein synthesis to specific regions [68] or microRNAs for use in modulating local translational activity. Asymmetric distribution of specific mRNAs in the cytoplasm was first visualized in the early 1980s when ISH techniques were used to detect β-actin mRNA in ascidian embryos [69]. Subsequent studies demonstrated that asymmetric mRNA localization contributes to the targeting of protein products involved in developmental processes, such as oocyte differentiation with the establishment of morphogenesis gradients [70–72] or early stage development of embryos [73–79].

**Figure 2.**
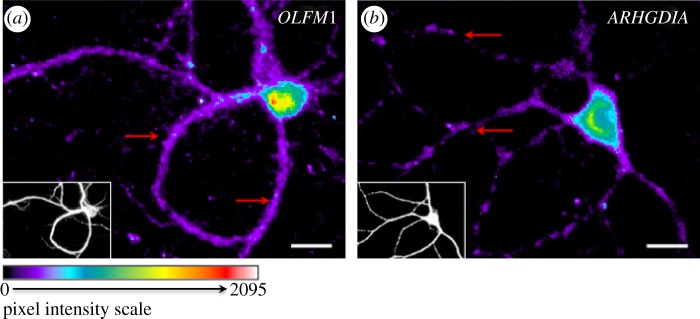
*In situ* hybridization reveals different patterns of localization in neuronal dendrites. Fluorescent microscopic evaluation of biotin-conjugated oligoprobes on paraformaldehyde-fixed 14-day-cultured mouse cortical neurons hybridized with biotin-conjugated 25mer-oligoprobes detected with streptavidin-Alexa568. For each image, the small bottom left corner panels represent MAP2 immuno-staining. Patterns of distribution are highlighted with red arrows. (*a*) Probe against *OLFM1* transcript illustrates a uniform distribution in dendrites; (*b*) probe against *ARHGDIA* transcript illustrates a punctated distribution in dendrites.

Single-cell studies are particularly useful in the central nervous system (CNS), given the high degree of cellular heterogeneity both among different cell types and within a single-cell type. In cultured neurons, FISH studies have highlighted the importance of mRNA localization. A large population of transcripts are confined in dendrites [80–82], and these transcripts are both locally translated [83–90] and spliced [91]. Remarkably, the subcellular distribution of transcripts is often non-uniform and punctate, suggesting that local translation is confined to ‘hot-spots’ [82,87,88,92,93] (figure 2). This subcellular regulation of transcript targeting and local translation allows for fast and accurate spatio-temporal control of cellular responses to environmental changes or synaptic input, and is critical for the establishment of synaptic plasticity [94]. Several dendritically localized transcripts have been described, and include structural proteins (MAP2), enzymes (CamK2a), growth factors (BDNF), ligand- or voltage-gated ions channels (glutamate and GABA receptor subunits; calcium channels) and transcription factors (CREB) [83,95–98].

## 8. Transcriptome profiling of single cells

**Figure 3.**
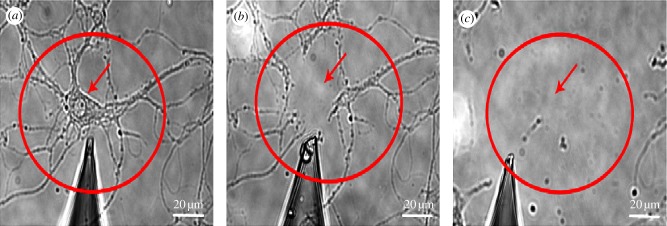
Mechanical severing of soma and dendrites from neurons. Rat hippocampal neuron in dispersed primary cell culture with its soma (red arrow) and dendrites (red circle) before (*a*) and after aspiration by a glass micropipette of the soma (*b*) and dendrites (*c*).

Single cell transcriptomics requires the isolation of RNA from the single cell and can be accomplished using a variety of methods the most common of which is by using a patch pipette (described in the electrophysiology section above) to puncture the cell and aspirate the contents (figure 3). This approach can also be used to isolate subregions of cells including neuronal dendrites as illustrated in figure 3.

Transcriptome analysis permits the analysis of multiple mRNAs simultaneously rather than one or a few at a time. Such analyses highlight the coordinate changes in gene expression that occur within cells, emphasizing an aspect of cell regulation that is missed in the analysis of one mRNA at a time. Several approaches can be taken to transcriptome profiling, including PCR, microarray and RNAseq, each of which has advantages and disadvantages.

Microarray has been the main high-throughput method for RNA profiling since its development in the mid 1990s. It is primarily used to study the global transcriptome in populations of cells. Before the development of microarrays (and even today), many laboratories relied on PCR to test expression levels of a small number of genes. However, PCR is limited by the restricted number of transcripts that can be studied simultaneously, and the exponential nature of transcript amplification can significantly distort the quantification, even with techniques such as quantitative PCR. Therefore, microarrays represent a substantial improvement over PCR. When samples are prepared using linear amplification [99], microarray provides quantitation of thousands of transcripts simultaneously from a single cell or from a population of cells. A microarray or DNA chip contains thousands of DNA probes that bind specific transcripts. Hybridization is quantified by labelling the amplified cDNA with fluorochromes and uses hybridization of cognate sequence between the RNA (or cDNA) and a predesigned cDNA probe (table 1).

**Table 1.** Comparison of RNA analysis techniques. In comparing the most common RNA analysis procedures with one another for use in single-cell transcriptomics, each procedure has advantages and disadvantages. The quantitation of signal with ISH is compromised by the permeability of the tissue while quantitation of qPCR is difficult because of the need to quantitate in the limited linear range of PCR amplification. Only RNA-seq does not require choice of probe for analysis and hence is the only procedure that is unbiased in the data that are generated. False-positives (calling a RNA present when it is not) arise in part from difficulty in controlling for specificity of detection methodology and only ISH can be selectively controlled so that no false-positives arise. Microarray gives rise to the most false-positives as sequence-specific hybridization hotspots are difficult to eliminate and control for. False-negatives (calling a RNA absent when it is not) arise most dramatically with ISH and qPCR, as specific sequences are needed to generate a positive signal, and if those sequences are incapable of binding to the probe (secondary structure, etc.), then no signal will be generated. False-positives and negatives for RNA-seq are negligible when performing paired-end 100 base reads but increase if doing shorter sequencing reactions (e.g. single-end 50 base reads).

	ISH	qPCR	microarray	RNA-seq
single-cell resolution	yes	yes	yes	yes
high-throughput	no	no	yes	yes
quantitative	with difficulty	with difficulty	yes	yes
unbiased	no	no	no	yes
cost	$	$$	$$$	$$$
ease of use	+++	+++	++	+
amount of data	+	++	+++	++++
false-positives	−	+	+++	+
false-negatives	+++	+++	++	+

The shortcomings of microarrays arise from poor control of stringency leading to cross-hybridizations, relatively low dynamic range and that quantification relies on use of pre-determined probes, potentially missing many previously uncharacterized transcripts. Microarrays have missed some important regulators of cell phenotypes in that an additional level of regulation is generated by isoforms and alternative splicing which are not easily detected using microarrays. Additionally, when many cells are combined and analysed by a microarray, there is an averaging effect where transcripts, only found in a few cells out of the isolated population, may fall below the detection limit, and thus, may not be recognized as a present transcript. The variability in expression profiles between single cells of the same type has now been shown a number of times, using microarray analysis. We are now at the point where intracellular variability is well recognized and should not be ignored.

To improve on the data obtained using microarrays, researchers have begun to benefit from the results obtained from high-throughput sequencing of RNA expression levels. The advent of mRNA sequencing eliminates the requirement to choose sequences for investigation, as there is no need to choose targets or probes. Sequencing can provide a greater depth of information regarding transcript variants and gives a more complete picture of the transcriptome, and, in turn, cellular phenotype. This advancing technology has been used to assess various biological questions, including adding a great deal of new information about transcript sequence variation in a short period of time [100] and has been used to produce a new paradigm for the identification of drug targets for pharmaceutical development [101,102]. Mapping of sequence reads back to the genome has revealed previously undetected splice forms of RNA, and examples of alternative exon use ranging from skipped sequences, cryptic sequences that are retained and combinations of specific sequences suggesting a form of exon selection/choice based on the presence of particular sequence patterns [103]. Variant splice forms detected are not limited to exonic coding region differences, as previously undescribed, retained introns have been found for a number of transcripts in various tissues [104]. The splicing, translation and ultimate function associated with these cytoplasmic intron-retaining transcripts are only beginning to emerge. Additionally, alternative 3′ and 5′ untranslated regions (UTRs) have also been identified in a number of tissues, and these forms may in fact be specific to those cell types or to particular cells of a given type with the tissue [105]. These features currently fall outside where their genes are thought to begin and end, and most likely would be discovered only by sequencing. These sequencing results are not entirely unexpected as previous deposits of transcript sequences are based on the most abundant isoforms present in cells, and are in turn mapped back to the genome to define what we think of as exons, introns, UTR and gene boundaries. While the first RNA sequencing studies involved sequencing library construction from bulk prepared RNAs, construction of libraries from aRNA has proved to be a robust and repeatable approach to assaying single-cell transcriptome by RNA sequencing. RNA sequencing promises to greatly increase the dynamic range, precision and resolution of RNA measurement. However, there are still several issues that require refinement, including removing biases from sequencing chemistry or computational processing.

An example of the type of problem that the single-cell transcriptomic's approach may be useful in investigating is to identify and quantify the mRNAs encoding the proteins involved in establishment of neuronal connectivity and therefore neuronal systems. Single-cell RNA-seq is ideally suited for this, as the pre-synaptic ligand-encoding gene neurexin and the post-synaptic-receptor encoding gene neuroligin can both undergo extensive alternative splicing, giving rise to cell-enriched forms of mRNAs and their translated proteins. The pairing of a selected neuroligin with its neurexin is critical in elaborating proper synaptic connections [106]. Another set of players in the CNS wiring schema are the protein products of DSCAM genes that encode cell surface IgG-like receptors that act as ‘hydrophobic repulsers’ of interactions, thereby inhibiting inappropriate synaptic connections [107]. Elucidation of which alternatively spliced forms, of each these gene products, are expressed in pre- and post-synaptic neurons will establish the wiring capability of these cells. Such wiring capacity is the key to establishing functional neuronal systems that underlie all aspects of neuronal functioning, and understanding how this is molecularly regulated will provide important functional and therapeutic insights into neurological and psychiatric illnesses. Such insights can be garnered only by using single-cell approaches.

## 9. Approaches to study gene expression in multicellular organisms *in vivo*

While some approaches to study the expression of multiple genes in multicellular organisms do exist, there is still not an ideal method that provides quantitative genome-scale data from a single brain cell *in vivo*. Such a method would prove valuable in several contexts. Combining genome-scale transcriptomics data with other physiological parameters would enable us to ask more directed questions towards understanding the underlying molecular regulation of complex neuronal functions such as learning and memory.

Progress towards this goal, however, has been made, and the level of cellular resolution largely depends on the rules of development in the respective cell lineages. In species with invariant and fixed cell lineages, such as the larva of *Caenorhabditis elegans*, automated digital expression atlases of gene expression have been generated using either ISH or fluorescent protein reporters [108]. Single-cell data can be generated from this simple organism because the cell lineages are largely invariant between individual worms, and thus, the same cells are easily annotated and identified by spatial discrimination. This approach, however, is limited when compared with global transcriptomic techniques, as this particular study only assessed 93 genes in 363 cells. And while it is an excellent model system in many contexts, it does not rival the complexity of cells in mammalian species where there are hundreds of millions more cells and cellular interactions.

ISH atlases have been generated for multicellular mammalian model organisms that do not have fixed cell lineages, such as mice. The Allen Brain Atlas provides a comprehensive genome-scale ISH atlas of the expression pattern of approximately 20 000 genes in the adult mouse brain [109]. Also, an embryonic midgestation ISH atlas investigated the expression of approximately 1000 genes in more than 90 distinct anatomical features [110]. In these datasets, the low channel capacity of *in situ* technology was complemented by computational techniques to merge the image data from multiple animals—thus, creating a synthetic multiplexed dataset. Furthermore, the Gene Expression Nervous System Atlas project provides an alternative approach to investigate gene expression using the BAC-transgenic mouse engineering system in which an EGFP and a polyadenylation site are introduced immediately upstream the ATG start codon of each gene. The hundreds of available BAC-transgenic mouse strains provide a valuable tool for tracking the spatial expression pattern of each gene in various brain regions, and to some extent, also provide information about cell type expression pattern, based on the morphology of EGFP-expressing cells. While these elaborate atlases are valuable tools for discovery-based investigations or as a reference for one's own gene expression data, none of them measure the simultaneous expression of multiple mRNAs in the same cell; hence the synthetic multiplexed datasets do not represent true covariation pattern among the mRNAs. As cellular function requires the coordinate expression of multiple genes, methods to simultaneously measure multiple RNAs from defined cells or cellular subregions will facilitate the discovery of these underlying modulators.

## 10. Approaches to study transcriptomics of single cells *in vivo*

Model systems that naturally occur as unicellular organisms or as single cells in suspension, such as bacteria, yeast and blood cells, have with relatively ease been used to investigate genome-scale gene expression in single cells [111,112] . Although we have learned a great deal about the regulation and timing of gene expression bursts in various contexts from these studies, the extrapolation of such data to the mammalian nervous system cannot be justified for many obvious reasons.

To overcome the challenges of brain tissue complexity, several experimental approaches have been used in the past, including laser capture microdissection (LCM) [100], fluorescence-activated cell sorting (FACS) [113,114], immunopanning (PAN) [114,115], translating ribosome affinity purification (TRAP) [116] and manual sorting (MS) [117] of reporter labelled cells from dissociated brain tissue. However, these methods, except for LCM, are not capable of combining isolation of RNA from single cells and collection of information about the specific location or physiological characteristic of the single cell in intact tissue. Although both the FACS and MS methods are capable of isolating single cells, neither of these can do so along with information about the cell's location in the intact tissue. While LCM does provide this information, it also has a downside. A comparative study of several *in vivo* RNA purification methods [118] found that LCM and TRAP contained significant levels of contaminating RNA when compared with FACS, PAN and MS. For instance, non-GABAergic cell samples contained significant amounts of GABAergic-cell-specific transcripts suggesting that these methods also capture a lot of unintended transcripts. Similar results were obtained for non-astrocytic samples containing astrocyte-specific transcripts. The same conclusion was drawn, when we did a similar comparative analysis of the TRAP and PAN data (D. Lovatt 2011, unpublished data). Given the structural complexity of brain tissue and that most cell types including neurons, astrocytes and vascular cells intermingle with one another, it is not surprising that LCM-derived profiles contain contaminating transcripts. The TRAP method theoretically is supposed to generate very clean profiles, but experimental evidence suggests that it also contains considerable contaminants. In this case, either unspecific expression of the TRAP construct or non-specific binding during the affinity purification step is the presumed cause of this contamination.

An ideal goal of single-cell biology would be to assay a complex mixture of molecules, e.g. the whole transcriptome, in individual cells within their natural tissue context. However, all of the existing methods have various limitations in this regard. There is no doubt that a novel approach, which in an unbiased way isolates mRNA from single cells *in vivo,* would provide a powerful tool to study how brain cells function coordinately to generate systems level physiologies, but also how cell types outside the nervous system elicit their specific biological functions and associations.

## 11. mRNA translation visualized in single cells *in vivo*

Cells have developed sophisticated mechanisms to dynamically respond to environmental perturbations and developmental programmes. The first level of regulation involves various well-known transcriptional and post-transcriptional regulation mechanisms as well as recently characterized novel mechanisms such as miRNA, antisense RNA and epigenetic transcription regulation. Cells also use post-translational regulation, which is much faster than transcriptional control and uses activation or deactivation of pre-existing proteins using kinases and phosphatases. This type of control is fast and does not require the synthesis of new materials, and is used widely by the cell to control signal transduction and enzymatic activities. For instance, the signalling pathways of G-protein-coupled receptors and the activation/deactivation of transcription factor Elk-1 [119–122].

Compared with transcriptional regulation and post-translational regulation, less attention has been focused on the translational regulation of mRNA. However, over the past decade, many studies have shown that the translational regulation is as important and sophisticated as transcriptional regulation, demonstrated by the use of modern high-throughput measurement technologies [123–125]*.* Schwanhausser *et al.* simultaneously measured absolute mRNA and protein abundances and turnover rates for more than 5000 genes in mouse fibroblasts showing that protein copy number is more correlated to translation rate than mRNA copy number. To this end, the coefficient of determination, *R*^2^, between mRNA abundance and protein copy number is 0.41, while *R*^2^ between translation rate and protein copy number is 0.95 [125]. They also showed that the influence of degradation of protein is minor.

Besides the global regulation mechanisms, the cell is known to have localized subcellular translation involving synthesis of new proteins at the specific subcellular sites where the protein is needed. This mechanism enables rapid replenishment of protein shortly after it is required without the need of transporting it from the soma to its final destination. Local translation is especially important in morphologically polarized cells, such as neurons, that have extremely long processes and need to control complicated synaptic activities in their distal processes. To show that local translation can occur, the existence of translational machinery, such as polyribosomes, translational factors and mRNAs in dendrites, has been demonstrated, and the dendritic transport mechanisms of some mRNAs have been elucidated [87,88,93,104].

**Figure 4.**
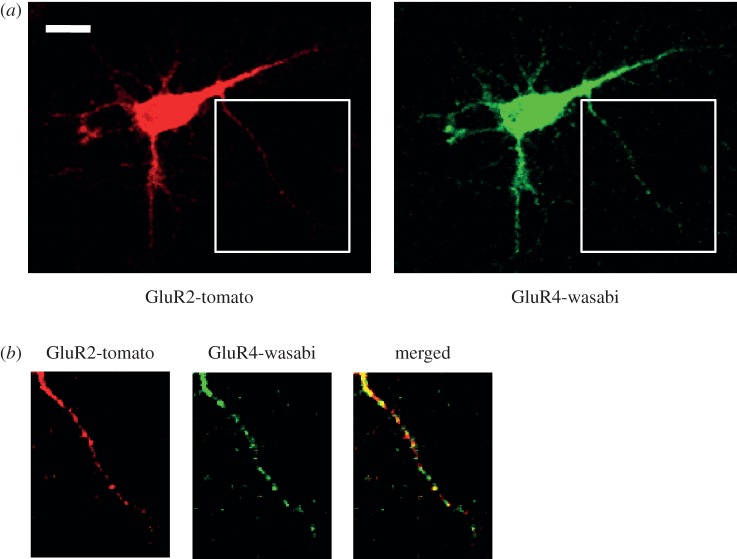
Live single-cell mRNA translation analysis. GluR2-tomato and GluR4-wasabi mRNAs when translated show distinct distribution patterns of translational hotspots in dendrites. (*a*) Fluorescent images of GluR2-tomato and GluR4-wasabi mRNAs transfected neuron. (*b*) Magnified images from insets from (*a*).

Local translation of dendritic mRNAs is known to be important in long-term synaptic plasticity, which modulates post-synaptic receptor abundances at synapses and therefore may be associated with underlying mechanisms of learning and memory [126]. Each neuron has many synaptic connections with multiple adjacent neurons, and each synaptic junction may require a different activity at different times. For this reason, centralized translation occurring in the cell soma is not an appropriate mechanism to fulfil the demands of each synaptic junction over time. Local dendritic translation shows different regulatory features when compared to translation in the cell body. Job & Eberwine [87,88] demonstrated the characteristics of local translation by transfecting green fluorescent protein-encoding mRNA into isolated dendrites. Local translation occurred in confined areas, called ‘translational hotspots’, and occurred quite rapidly while translation in the cell body was much slower [87,88]. Moreover, individual translational hotspots on the same dendrite showed different kinetics after the translational stimulation by adding the metabotropic glutamate receptor agonist dihydroxyphenylglycine (DHPG). There was no apparent correlation between the position of the hotspot and its translational pattern. However, this observation suggested that each hotspot responded differently to the external perturbation. To further explore this observation, we co-transfected two different glutamate receptor subunit mRNAs (Gria2 and Gria4 mRNAs) tagged with green or red fluorescence protein modules and observed local dendritic translational activities of two mRNAs by measuring green and red fluorescent signals. Translational hotspots of Gria2-RFP and Gria4-GFP overlapped in many areas but, in some areas, they were shifted and even offset each other (figure 4). When we observed the local translational activities over time, translational hotspots of two mRNAs showed different kinetics, which suggested that local translational regulation is not only regulated by site-specific control, but can also discriminate two different dendritically localized mRNAs.

As exemplified earlier, cells show dynamic changes in their molecular state at various timescales. One of the fastest timescales is post-translational modification but recent evidence seems to suggest potential for rapid dynamics involving localized translation. Work in our laboratory suggests that localized translation seems to involve both site-specific and mRNA-specific dynamics. Thus, cells show complexity not only at the single-cell level but within subcellular compartments of the same cell. One of future goal of single-cell analysis is to understand the regulatory control mechanism for such fine-scale translational dynamics.

## 12. mRNA regulation by RNA-binding proteins in single cells

RNA-binding proteins (RBPs) are integral to all aspects of RNA biology, including the regulation of gene expression at the level of pre-mRNA processing, export, localization, stability and translation. All of these physiological events are tightly controlled by a series of interactions with myriad RBPs complexed as multi-megadalton ribonucleoprotein (RNP) particles [127,128]. Many of these interactions are transient as well as motile, as exemplified by the final step of translation, translocation, which requires coordinated movement of tRNAs, mRNA, and the 30s and 50s subunits of the ribosome [129]. RBPs often contain multiple, highly conserved RNA-binding sequence motifs that are combined in a modular fashion. This allows for a number of diverse weak interactions that combine to generate highly selective or less-specific interactions, conformational variation and extensive protein–protein interactions [130]. RBPs influence the complexity of the RNA population as well as the spatial and temporal expression of each transcript within each of the various compartmentalized domains of a single neuron.

Characterization of RNA–RBP interactions is crucial to understanding the dynamic processes that encompass RNA biology, and methodologies are needed that will allow for mapping of these interactions. In the past decade, systematic efforts to identify the full complement of cell-specific mRNA transcripts have been aided by advances in highly paralleled platforms that have been critical for to identifying native mRNAs as well as their respective alternatively spliced [131–133], differentially polyadenylated [134] or RNA-edited variants [135]. However, a more complete functional understanding of mRNA regulatory networks requires a catalogue of RBP-target mRNA interactivity and the precise mapping of the target mRNA footprint that underlies the strength of its high-affinity binding. Modelling of bona fide target sequences suggests that mRNAs contain functional sequence motifs having a defined secondary structure that stably assembles at a lower free energy of formation than expected by random chance [136,137]. As a result, many *in silico* prediction algorithms incorporate such a screen for functional mRNA sequences [138,139].

There are two general approaches to studying RNA–RBP interactions: targeting a known RBP to identify the RNAs that interact with it, or targeting a specific RNA to determine which RBPs bind to it. In the former category, common analytical methods are the gel-shift or supershift assays where the mobility of a RNA in a gel is hindered, or ‘shifted’, by its interaction with a RBP or RBP–antibody complex, respectively. Other various selection procedures have been used in the past to identify RBP-associating transcripts, including iterative RNA aptamer methods (i.e. SELEX) [140,141] and immunoprecipitation of RNP complexes followed by RT-PCR on a few target RNAs, or  microarray analyses or high-throughput sequencing for a more global screening. All of the methods described earlier are *in vitro* methodologies and as such they are limited in their ability to reflect the scope and dynamic nature of RNA–RBP interactions that occur in living cells under varying conditions and stimuli. This is reflected in the technical constraints of these enrichment strategies, which encumber them with limited scalability, relatively narrow dynamic ranges of resolution, low signal-to-noise ratios and difficulties in separating direct versus indirect associations. For example, one consistent criticism of immunoprecipitation techniques is the potential for false-positives due to the fact that the lysis step allows all the neuronal constituents to mix. As a result, RBPs and mRNAs normally compartmentalized within separate neuronal domains may be allowed to interact in a non-physiological manner. Thus, methods are needed that will more accurately reflect RNA–RBP interactions under physiological conditions. A more optimal approach also necessitates identifying these interactions within an *in vivo*-configured RNP complex.

**Figure 5.**
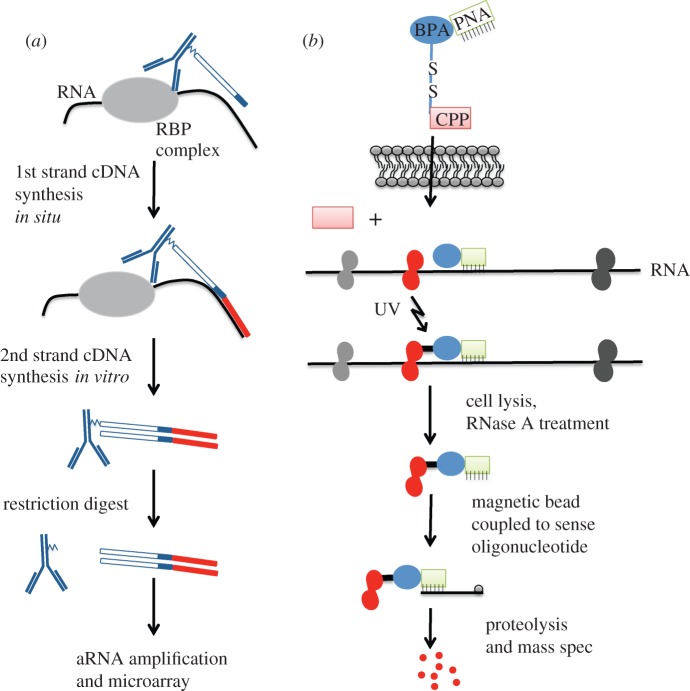
Antibody-positioned amplification and PNA-assisted isolation of RNA-binding protein technologies. (*a*) Schematic of the APRA method. Antibodies conjugated to an oligo (blue) are applied to fixed and permeabilized cells from primary cultures. Association of the antibody with the RBP positions the oligonucleotide in close proximity to RNA interacting with the RBP. First-strand cDNA synthesis is performed *in situ* using a degenerate nucleotide sequence at the end of the oligo (dark blue band). Red bar indicates newly synthesized cDNA. The complexed antibody–DNA is then removed from the cells, and second strand synthesis is performed *in vitro*. The antibody is removed from the double-stranded DNA by restriction digest. The cDNA can then be used for aRNA amplification and microarray analysis. (*b*) Schematic of the PAIR method. The PAIR peptide contains a CPP (red), which allows the peptide to enter the cell. Once the cell membrane is crossed, the BPA(blue)-PNA(green) complex will dissociate from the CPP and hybridize to complementary sequence on target RNA. Application of UV irradiation cross-links the RBP (red) in near proximity to the BPA. Cells are then lysed and RNase treated. PNA–RBP complexes are isolated using sense oligonucleotides coupled to magnetic beads. This material is proteolysed and further processed for mass spectrometry.

Antibody-positioned RNA amplification (APRA) was developed to overcome these limitations [142] (figure 5*a*)*.* In this technique, a priming/amplification oligonucleotide is covalently coupled to an antibody recognizing an epitope on a specific RBP. Using fixed and permeabilized primary neurons dispersed in culture or brain sections, the antibody is allowed to bind to the RBP of interest and positions the priming oligonucleotide in proximity of mRNAs sequestered within the RNP complex. The priming oligonucleotide contains a 15 nucleotide degenerate sequence at its 3′-end, which allows for the *in situ* transcription of mRNA sequences not masked by the RNP complex. Following first-strand cDNA synthesis, the antibody–cDNA complex is removed, isolated and converted into a double-stranded cDNA. The presence of a T7 RNA polymerase promoter incorporated into the priming oligonucleotide allows the target mRNA sequences to be further amplified before being analysed by microarrays or next-generation sequencing. As proof of concept, this strategy was applied to the identification of mRNA cargoes for the *Fmr1* gene product, the fragile X mental retardation protein (FMRP). Using primary hippocampal cultures, an initial FMRP APRA screen resulted in 223 putative positive targets using an approximately 1100 cDNA macroarray. Of these putative targets, a subset of 83 were selected for secondary screens consisting of filter binding and UV cross-linking studies to confirm the presence of a direct interaction with FMRP. Of these 83 APRA positives, approximately 73 per cent (61/83) were also positive in the filter binding assay when compared with control, and 82 per cent (50/61) of the filter binding assay positives also showed UV cross-linking. These data show that the APRA methodology was quite robust at identifying mRNA cargoes of FMRP even within an *in situ* RNP complex containing numerous other RBPs and mRNAs. As the APRA-associated mRNAs are linearly amplified for analysis, the method can be applied to single cells.

Another *in vivo* procedure was developed by Darnell and co-workers [143,144], in which the UV cross-linking of triturated live cells or plated primary cultures was coupled with stringent immunoprecipitation methods to isolate targets of proteins showing RNA-binding activity. This technique attempted to exploit the intrinsic photo-reactivity of nucleic acid pyrimidine base pairs and some amino acids, most notably the aromatic residues found in proteins, to covalently couple RNP components prior to isolation and identification. However, a multitude of non-specific interactions have been generated with this approach, requiring careful secondary screening to eliminate false-positives.

The PAIR approach, peptide nucleic acid (PNA)-assisted identification of RNPs [145], was developed to enable the identification of RBPs interacting with an RNA of interest *in vivo* (figure 5*b*). In this methodology, a PNA complementary to the target RNA is coupled to a cell penetrating peptide (CPP), and a photoactivatable compound. The CPP, transportan 10 (TP10), carries the molecule into the cell and detaches from the PNA after entry, as the disulphide bond between the CPP and the PNA is reduced. The PAIR PNA then binds to its complementary RNA in the cytoplasm. The PNA–RNA bond is strong and specific, as the PNA backbone contains no charged phosphate groups to generate interference, and has a highly flexible structure. When exposed to UV irradiation, the benzoyl moiety of the photoactivatable amino acid adduct, *p*-benzoylphenylalanine (Bpa), is released and the newly generated free phenylananine radical is then able to cross-link molecules in close proximity, thus capturing RBPs binding to or found in complex with the RNA target. For UV cross-linking to occur, the distance between the two moieties has to be 4.5 Å or less [146], thus the RBPs found are likely to bind directly to the RNA in the region of PNA binding or to be part of a multiprotein complex in the immediate vicinity. After UV cross-linking, the cells are lysed and the PNA–RBP complexes are isolated using streptavidin magnetic beads coupled to a biotinylated sense oligo that recognizes the PNA sequence. Identification of RBPs is then accomplished by excising bands from a SDS/PAGE gel, followed by trypsin digestion and mass spectrometry (MS).

Many of the regulatory sequences of an mRNA are located in the 5′ and 3′ UTRs. We used PAIR to probe for RBPs that bind to three different regions in the 5′ and 3′ UTRs of *ankylosis* (*ank*) mRNA, a dendritically localized transcript globally expressed in all neurons. Using PAIR, 23 known RBPs interacting with *ank* mRNA were identified [146]. Among them, some (nucleolin) were found to bind to all regions targeted under all physiological conditions tested (basal, BDNF, K+ and DHPG treatments), while other interactions were limited to a specific region or experimental condition. Curiously, more similarities were found between binding patterns of PNA3 (located in the 5′UTR) and PNA2 (located in the 3′UTR) than between PNA2 and PNA1, which are located within a few bases from each other in the 3′UTR. This might be the result of separate, unrelated binding events at each site, but it is also possible that the binding pattern is indicative of the transcript folding in a way that brings the PNA3 and PNA2 binding sites in the 3′ and 5′UTRs, respectively, in a close proximity to each other, which might be a mechanism of translational control [147]. The specificity of the PNA binding and the short distance required for UV cross-linking will make PAIR a highly targeted tool for RBP discovery. Thus, the interactions we have revealed for *ank* mRNA are likely to be a subset of all of its RBPs interactions. To expand its scope, more PNAs would need to be synthesized along the length of the transcript. The results we obtained by PAIR well capture the complex picture of the varying types of interactions that occur between RNA and RBPs: some are more universal in nature; others are highly specific and dynamically modified in response to environmental stimuli.

## 13. Single-cell peptidomics/proteomics

While we have had the ability to assess a limited number of proteins in single cells using immunocytochemical approaches, the analysis of the protein complement (genomic scale) in single cells is more difficult than examining the transcriptome. The reason for this difficulty is the chemical composition of proteins that are made up of 22 amino acids, some of which can be chemically modified, thereby creating peptides and proteins that are differentially charged and structurally complex. This is in contrast to RNA, which has only four nucleotides and is always negatively charged (still can be structurally complex). The standard single-cell issues of molecule separation and detection become even more difficult with such chemically complex molecules.

In an effort to deal with these issues, various approaches are being explored for peptide/protein analysis. In early studies, Sweedler and colleagues used matrix-assisted laser desorption ionization (MALDI) MS to characterize a subset of proteins from single *Aplysia* neurons [148,149]. This animal species model system has the advantage of analysis of large cells (approx. diameter of 60 μ) containing a high abundance of selected peptides. This approach is complemented by the work of Dovichi and co-workers where they used capillary zone electrophoresis to essentially perform gel separation of proteins based upon the charge-to-mass ratios of the cellular proteins [150,151]. This approach works because of the small volume of the capillary zone tubes, thereby allowing the cellular proteins to be concentrated in a small volume, resulting in a higher protein concentration for analysis. Decreasing the volume of sample and surface area to which the sample is exposed (which causes losses) is being explored through the development of Nano-LC–MS/MS which promises to increase standard MALDI sensitivity by two to threefold [152].

A recent ingenious effort to increase the sensitivity of MS methods for protein detection called stable isotope labelling by amino acids in cell culture (SILAC) invokes the use of heavy stable, non-radioactive isotopes, such as C13 (arginine for example) to *in vivo* label proteins in one cellular sample [153,154]. The labelled proteins are isolated and MS analysed at the same time as a protein sample from a non-C13 labelled sample. All peptide fragments with labelled arginine are shifted in their mass by six units (for single arginine-containing fragments). This difference in mass is easily detectable and has provided a more sensitive approach to protein detection. There are currently several laboratories attempting to use C13 and N15 SILAC to analyse the protein complement of single cells. This may be enhanced by the use of Nano-LC–MS/MS, where small amounts of starting material are presented to the MS lasers in a manner that makes them more volatile producing a higher yield of products.

Other single-cell proteomics approaches are focused around the use of antibodies to specifically capture proteins. One early example of this is termed immuno-detection amplified by T7 (IDAT) in which antibodies to specific proteins were covalently attached to a double-stranded cDNA containing a T7 RNA polymerase promoter site [155]. The antibody–antigen interaction is detected by highly sensitive nucleic acid amplification. This has also been done using complementarity determining regions (CDRs) rather than antibodies [156,157] and using PCR rather than T7 RNA polymerase [158].

Another version of antibody detection of the single-cell proteome that does not require amplification is to use TIRF microscopy to assess antibody–protein interactions. In a recent example of this, single cells are separated into individual chambers using optical tweezers after which, the cells are lysed by laser microcavitation and the liberated proteins are moved into a chamber in which selected antibodies have been microprinted. Selected proteins from the single cell bind to their corresponding antibodies and are detected and measured by TIRF [159]. Protein specification comes from the identity of the antibody in the microprinted spot. Background binding of antibodies is one of the most severe limitations of this approach, decreasing its sensitivity to detection of hundreds of proteins.

At the moment, the quantitation of the total single-cell proteome is beyond our technical capabilities. However, the limited single-cell proteomics abilities that we currently have do enable selected questions to be addressed quantitatively. Hope for resolving these technical issues comes from the fact that only a few years ago it was impossible to assess the single-cell transcriptome, which is now readily doable.

## 14. Conclusions

Recent developments in measurement technology are providing increasingly higher resolution information of the complex molecular dynamics of a cell, both in single-cell organisms and in multicellular organisms. As always, higher resolution information leads to significant advances in applications. For example, by not losing information to averaging, our understanding and inference of complex pathway dynamics or network interaction of molecules are enhanced. In fact, by providing information about individual molecular interactions within each cell, single-cell functional data may fundamentally change our understanding of molecular process control. On the more practical side, having single-cell resolution data will help increase our ability to discern important molecular therapeutic targets in two important ways. First, being able to isolate phenotypically or developmentally distinct subpopulations of cells (e.g. immediate derivatives of stem cells) and carrying out a genome-wide unbiased assay of molecular differences will allow us to identify key molecular players in a more efficient manner. That is, by assaying cells with very small molecular differentiation at a very high resolution, we will be able to more directly identify key functional differences, in contrast to broad and noisy inferences that result from tissue-level assays. Second, it is often the case that responses to therapeutic agents can be variable at the single-cell level, which is also reflected in heterogeneous patterns of cell dysfunction in diseases. Therefore, having single-cell resolution of responses to therapeutic manipulations will help identify subpopulations of differentially affected cells and help eliminate both false-positive and false-negative results.

It is clear that higher resolution information from single-cell measurements will lead to more refined inferences. However, the emerging data on variation and complexity at the single-cell level raise some fundamental questions about the biology of individual cells. One question examined earlier is the functional and fitness consequences of cell-to-cell variability. We discussed before the idea of ‘bet-hedging’ in single-cell organisms as a life-history strategy mediated by switching individual cells to different phenotypic states. It is also possible that tissue-level function in a multicellular organism requires single-cell diversity. The most obvious case is the known functional diversity in sensory neurons, but we can easily imagine functional requirements for single-cell diversity for the CNS or other organs. On the other hand, known developmental diseases arising from fluctuating variation demonstrate the negative consequences of variation. The existence of many complex genetic circuits, especially the curious proliferation of paralogous genes with redundant function, has been suggested to be due to their function in mediating canalization of the variation.

What is required is a theory of cell phenotype at the level of individual cells and their integrated function in tissues. Future work will need to map out molecular single-cell variability associated with cell phenotypes, ideally in real-time over the molecular and phenotypic dynamics. Our grand hope is that with sufficient data at the resolution of individual cells, we will be able to develop a theory of control of molecular variation for each cell and a theory of functional phenotypes that result from any given molecular state.

The quantitative analysis of single-cell biology initiated 350 years ago is becoming ever-increasingly possible, with the discovered complexities providing unprecedented insights into cellular function. Indeed, such insights highlight the fact that when studying the smallest unit of cellular systems (the single cell), the ‘orchestration’ of cellular constituent interactions is the basis of cellular identity and function. Understanding this orchestration and its complexities in single cells promises to provide the ‘score’ that underlies the development of cellular systems, tissues and organisms.
